# Distribution, Contents, and Health Risk Assessment of Cadmium, Lead, and Nickel in Bananas Produced in Ecuador

**DOI:** 10.3390/foods8080330

**Published:** 2019-08-08

**Authors:** David Romero-Estévez, Gabriela S. Yánez-Jácome, Karina Simbaña-Farinango, Hugo Navarrete

**Affiliations:** Center for Applied Studies in Chemistry, Pontificia Universidad Católica del Ecuador, CESAQ-PUCE, Ecuador, Av. 12 de Octubre 1076 y Roca, Quito, Pichincha 17012184, Ecuador

**Keywords:** atomic absorption spectrophotometry, exportation product, food contamination, graphite furnace, health risk, heavy metals, *Musa* sp.

## Abstract

In this study, cadmium (Cd), nickel (Ni), and lead (Pb) contents were analyzed in sixteen banana composite samples from different commercial establishments from eleven Ecuadorian production provinces using graphite furnace atomic absorption spectrophotometry. The concentrations (fresh weight) in the samples collected (9.3–47.3 μg·kg^−1^ for Cd, 16.1–105.6 μg·kg^−1^ for Ni, and 36.9–538.0 μg·kg^−1^ for Pb) were used to calculate the estimated daily intake (EDI), target hazard quotient (THQ), and target carcinogenic risk (CR) associated with dietary exposure to these potentially toxic metals. Cd and Ni results showed that every sample had EDIs lower than the oral reference dose and THQ values lower than 1, demonstrating that there was no non-carcinogenic risk related to the exposure to Cd and Ni. In the case of Pb, two EDIs results were higher than the reference dose, also their corresponding THQ values were higher than 1. The lead CR in all samples was less than 1 × 10^−4^, the upper limit used for acceptable cancer risk. Thus, there is no significant health risk to the consumer associated with bananas with contamination levels of Cd, Ni, but there is Pb risk for toddlers (12 kg of body weight) intake comparable to the one detected in the present study.

## 1. Introduction

Fresh fruits like bananas (*Musa* sp.) are important to the human diet because of their vitamin and mineral salts contents. However, they may also contain toxic metals [1].

In recent years, environmental contamination from heavy metals has been a worldwide concern because of heavy metals’ persistence, their ecological risks, and their mobility between biotic and abiotic spheres [2]. The increasing risk of human non-occupational exposure is related to the direct contamination of food [1,3,4,5,6]. Cultivated products are of greatest concern owing to their direct contact with environmental contaminants, and they are also the first contributors to the food web [7]. “Dietary intake of plant-derived food represents a major fraction of potentially health-threatening human exposure” [8]. Heavy metals’ presence in cultivated food products depends on many factors, principally on the natural soil composition, environment, genotype of the plant, fertilizers, and/or metal-containing pesticides [9].

Not all heavy metals are unsafe for humans. Some are classified as essential for human metabolisms, such as copper, zinc, iron, manganese, selenium, and cobalt, whereas other metals are considered as probably essential, such as vanadium, and others such as arsenic, cadmium (Cd), lead (Pb), mercury, and nickel (Ni) are categorized as toxic [10]. 

The excessively high intake of toxic metals by humans and animals is dangerous, and the bioaccumulation of these metals has been reported to have carcinogenic, mutagenic, and teratogenic effects [1,8,11]. It has also been established that more than 95% of the total daily exposure to toxic metals comes from the ingestion of contaminated food [9].

The International Program on Chemical Safety (IPCS) and the International Agency for Research on Cancer [12] has considered classifying Cd as a carcinogenic substance (Group 1) as there is a high probability that it can cause renal effects, calcium metabolism disorders, hypertension and cardiovascular disease, and cancer, among others [13,14]. Pb, similarly to Cd, produces progressive toxicity in humans [15]. This metal causes health disorders such as renal dysfunction, spontaneous abortion and reduced birth weight, and affections in the immune system. Pb is also classified as a probable carcinogen to humans, Group 2A [16,17]. Both Cd and Pb have damaging effects on humans and animals because no effective mechanism exists for their elimination [9]. In the case of Ni, it has not been considered as lethal as Cd and Pb, but in spite of this is classified as a probable carcinogen to humans, Group 2A. When Ni exceeds the toxic concentration levels, it may produce pathological pulmonary lesions, including hemorrhage, edema, and cellular derangement [18]. It also affects the liver, kidneys, adrenal glands, spleen, and brain [19,20]. 

The European Commission Regulation [21] has established maximum levels for some contaminants, including heavy elements like Cd and Pb, of 50 μg·kg^−1^ and 100 μg·kg^−1^ in fruits, respectively. The Food and Agriculture Organization of the United Nations (FAO) for the World Health Organization (WHO), in the International Food Standards (CODEX 193), have established threshold values as 100 μg·kg^−1^ for Cd and Pb in different types of natural products [22]. In the case of Ni, neither the European Commission Regulation nor the FAO have established the threshold values, nevertheless the Environmental Protection Agency’s Integrated Risk Information System (EPA-IRIS) has established it as 300 μg·kg^−1^ [18]. In addition, the FAO has also established the CODEX STAN 205 [23] specific for banana, which refers to the contaminant limits cited in the CODEX 193.

Referring to the health risk assessment, the EPA has established oral reference doses (RfD) of 1.0 and 20.0 μg·kg^−1^·d^−1^ for Cd and Ni (soluble salts), respectively [24]. There is no EPA RfD value for Pb, but the United States Food and Drugs Administration (US FDA) Interim Reference Levels for lead is 3 μg·day^−1^ for children and 12.5 μg·day^−1^ for adults [25]. The European Food Safety Authority has mentioned the hazard reference value for Cd is 2.5 μg·kg^−1^·body weight^−1^·week^−1^ (2013), for Ni is 2.8 μg·kg^−1^·body weight^−1^ (2015), and for Pb is 25 μg·kg^−1^·body weight^−1^·week^−1^ (2005) [26].

Bananas are among the five most important food crops in the world, and Ecuador is a leading banana producer and exporter [27]. According to the Central Bank of Ecuador, bananas are the second-highest non-oil export product, with a generated revenue of 3.196 million USD in 2018 [28]. Banana exports represent 2% of the country’s general gross domestic product (GDP) and approximately 35% of its agricultural GDP [29]. 

Although there are a number of studies in which banana peels have been used as a material for remediation and uptake of contaminants [30,31,32], published information about Ecuadorian bananas’ heavy metal content is limited [27,33]. Therefore, it is important to examine the presence of toxic metals in Ecuador’s natural foodstuffs, not only to be able to control them in accordance to the defined maximum residue levels set by authorities, but also to constantly monitor and compare those levels with data available in the literature in the absence of such limits [9]. Additionally, the WHO, through its Global Environmental Monitoring System/Food Contamination Monitoring and Evaluation Programme (GEMS/Food), is guiding and supporting countries such as Ecuador in the execution of research in food contaminants to determinate dietary exposure to chemical contaminants [9].

In this sense, the aim of this study was to (i) determine concentrations of Cd, Ni, and Pb in banana samples collected from various banana producing provinces in Ecuador, (ii) calculate the estimated daily intake (EDI) of Cd, Ni, and Pb associated with the consumption of Ecuadorian bananas, and (iii) determine the carcinogenic and non-carcinogenic risks of ingesting Ecuadorian bananas for toddlers, children, and adults. 

## 2. Materials and Methods

### 2.1. Sample Collection and Preparation Process

Sixteen composite banana samples were collected from eleven banana-producing provinces: Azuay, Bolívar, Cañar, Cotopaxi, El Oro, Esmeraldas, Guayas, Los Ríos, Manabí, Santa Elena, and Santo Domingo de los Tsáchilas. Each sample comprised five subsamples of specimens purchased in randomly chosen local commercial establishments within the sampling locations. The samples were immediately placed in plastic bags, and the air inside the bag was removed using cold-water immersion and the bag then sealed. The samples were stored on ice to delay the fruits’ oxidation process for 48 hours.

The samples were washed with high-quality reagent water (resistivity 18.2 MΩ·cm^−1^) to eliminate impurities. The peeled samples were mashed and homogenized to obtain the composite sample.

The water content of the composite samples was determined using a humidity analyzer (Mettler Toledo, HB43-S, Greifensee, Switzerland). Then, the composite samples were dried for approximately 24 hours at 70 °C in a Memmert UM 500 stove (Schwabach, Germany) until a constant weight was achieved. 

An approximately 1.0000 g sample was weighed in Teflon vials, then 5 mL of 70% nitric acid (Fisher Chemical, Certified ACS plus, CAS# 7697-37-2, Fair Lawn, NJ, USA) and 3 mL of 30% hydrogen peroxide (Fisher Chemical, Certified ACS plus, CAS# 7722-81-1) were gently added. Acid digestion was performed using a MARS 6 microwave (CEM, Matthews, NC, USA), taking as reference the analytical method IPN AC-06-00 [34], modified and verified for its applicability for the chemical analysis of metals in biological matrixes. 

### 2.2. Metal Determination

All the digestions of the composite samples were filtered, and then Cd, Ni, and Pb were analyzed using a graphite furnace absorption spectrophotometer (HGA 900 and AAnalys 400, Perkin Elmer Inc., Whaltham, MA, USA). Calibration curves were prepared using four concentration levels of dilutions of certified reference materials of 0.5, 1.0, 2.0, and 4 μg·dm^−3^ for Cd, and 5.0, 10.0, 20.0, and 40.0 μg·dm^−3^ for both Ni and Pb. Linear regression coefficients (*R*^2^) higher than 0.99 demonstrate linear adjustment between concentration and absorbance.

The standards of the calibration curve, samples, and blanks were prepared using analytical grade reagents and high-quality reagent water. Sample analysis was performed in triplicate using fortifications of known concentrations as quality control. The results are presented in μg/kg of dry weight.

The results obtained were evaluated against the corresponding threshold values 50 μg·kg^−1^ for Cd [21], 300 μg·kg^−1^ for Ni [18], and 100 μg·kg^−1^ for Pb [21,22].

### 2.3. Quality Control

To ensure the reliability and performance of the mineralization technique and the quantification method, the rates of standard deviation (RSD) and the accuracy as recovery rates of fortifications were also evaluated using the criteria established by the Association of Official Analytical Chemists [35]: Precision of 8% for repeatability and recoveries between 75% and 120% for accuracy.

For the fortifications, known concentrations of certified reference materials of approximately 1000 mg/L were added to original non-fortified samples. All the samples were fortified according to each metal’s quantification limit level: 12.5 μg·kg^−1^ for Cd and 125 μg·kg^−1^ for both Ni and Pb. The standards used were:

- Cadmium Certified Reference Material Certipure^®^ (Merck, Darmstadt, Germany), 986 mg/kg ± 4 mg/kg, density 1.0131 g/cm^3^, Ord. No. 1.19777.0100, Lot No. HC60709577.
- Lead Certified Reference Material (Inorganic Ventures, Christiansburg, VA, USA), 999 μg/cm^3^ ± 3 μg/cm^3^, density 1.010 g/cm^3^, Cat. No. CGNI1, Lot No. J2-NI02103.
- Nickel Certified Reference Material (Inorganic Ventures), 1003 μg/cm^3^ ± 5 μg/cm^3^, density 1.002 g/cm^3^, Cat. No. CGPB1, Lot No. M2-PB656988.

### 2.4. Human Health Risk Assessment

The human health risk assessment was evaluated based on the EDI and the target hazard quotients (THQ) for non-carcinogenic and carcinogenic risks. In addition, the recommended values for banana intake were determined. All these parameters were calculated using each concentration of toxic metal determined in the samples as well as different body weights: 12 kg for toddlers (aged 1–3 years) [36], 25 kg for children (aged 5–10 years) [37,38,39], and 60 and 70 kg for adults [36]. The daily intake sample amount was the mean weight of the banana samples (110 g). All the calculations were based on the EPA formulas [40,41,42].

#### 2.4.1. EDI

First, the EDI values as the chronic daily intake (expressed in µg·kg^−1^·day^−1^) were calculated using the EPA [40] exhibit 6–18 equation as follows:

(1) $$EDI=\frac{C\times IR\times FI\times EF\times ED}{BW\times AT},$$

where C is the concentration of each metal in the samples (expressed in µg·kg^−1^); IR is the ingestion rate (the mean weight of samples 0.110 kg per day); FI is the fraction ingested from contaminated food (1, unitless); EF is the exposure frequency (365 days per year); and ED is the exposure duration (ED, 70 years). These are in relation to body weight (BW, kg) and the averaging time (AT, ED × 365 days per year). 

#### 2.4.2. Non-Carcinogenic Risk

The potential health risks of contaminants were estimated using THQ. The THQ values were calculated using the EPA [42] exhibit 1–3 equation, as follows:

(2) $$THQ=\frac{EDI}{RfD},$$

where EDI is the chronic daily intake (expressed in µg·kg^−1^·day^−1^), and the EPA RfDs are 1.0 and 20.0 µg·kg^−1^·day^−1^ for Cd and Ni, respectively [24]. There is no EPA RfD value for Pb, but the United States Food and Drugs Administration (US FDA) Interim Reference Level for lead is 3 μg·kg^−1^·day^−1^ for children and 12.5 μg·kg^−1^·day^−1^ for adults [25]. 

THQ values lower than one (1) indicate that consumers are unlikely to experience any adverse health effects. If the THQ value is equal to or higher than one, there is a potential health risk.

The total cumulative health risk (TTHQ) was calculated by adding each metal’s THQ using the [41] formula as follows:

(3) TTHQ=THQ(Cd)+ THQ(Ni)+THQ(Pb).

For the evaluation, a greater TTHQ value means a greater level of concern.

#### 2.4.3. Carcinogenic Risk 

Carcinogenic risk (CR) is equivalent to the increased probability of an individual developing cancer over his/her lifetime due to exposure to the metals included in this study. Lead’s CR was estimated in accordance with the existing slope factor (SF) provided by the EPA [24], whereas Cd and Ni do not have SF values, thus CR could not be estimated. The following EPA [42] exhibit 1–3 equation was used:

(4) CR=SF×EDI,

where SF is the carcinogenic slope factor of 0.0085 (mg/kg/day)^−1^ for Pb, and EDI is the estimated daily intake of heavy metals (expressed in mg·kg^−1^·day^−1^).

CR values lower than 10^−6^ were considered negligible, values between 10^−6^ and 10^−4^ were considered within an acceptable range, and values higher than 10^−4^ were considered intolerable [41,42].

## 3. Results and Discussion

### 3.1. Sample Collection and Preparation Process

In the first stage of this study, three composite samples from the provinces of Los Ríos and Guayas and two composite samples from El Oro were used, as they were considered the most representative provinces in terms of banana production [43]. For each of the other provinces, just one composite sample was used. The specimens were purchased in randomly chosen commercial establishments located near production zones and that sold bananas for different producers. The subsamples were classified, the composite samples were assembled, and the water content was determined in all the samples. The water content ranged from 65.55–73.66% (average 71.17%). 

### 3.2. Metal Determination

After the sample preparation process, the concentrations of Cd, Ni, and Pb were determined. The linear regression coefficients (*R*^2^) were 0.997859, 0.999854, and 0.996738 for Cd, Ni, and Pb respectively, and in all cases higher than the expected 0.99, showing the linear adjustment between concentration and absorbance in the ranges of determination.

Each sample and its corresponding fortification were analyzed in triplicate. The results of the samples and their quality controls are shown in Table 1. The concentration ranges found were between 9.3 μg·kg^−1^ and 47.3 μg·kg^−1^ for Cd, between 16.1 μg·kg^−1^ and 105.6 μg·kg^−1^ for Ni, and between 36.9 μg·kg^−1^ and 538.0 μg·kg^−1^ for Pb.

All the calculations were done using Microsoft ® Office Excel 2016 (Microsoft Corporation, Redmond, WA, USA).

Both Cd and Ni results were lower than the threshold values established in both European Commission regulation [21] and the EPA-IRIS [18], respectively. For the Pb results, four of the sixteen locations studied were lower than the respective recommended threshold values for European Commission Regulation [21] and CODEX 193 [22]. The higher results were from the samples collected in Bolivar and Santa Elena provinces, which had 420.9 μg·kg^−1^ and 538.0 μg·kg^−1^ of Pb, respectively, approximately four and five times the 100 μg·kg^−1^ CODEX threshold value. The lowest values obtained were in samples from Azuay province (36.9 μg·kg^−1^). 

No previously published studies focusing on Ecuador exist to which this information can be compared. Nevertheless, Felix et al. [44], in a study related to the concentration of toxic metals in agricultural soils, found that the Pb content in sampled banana soils corresponded to a maximum value of 5.36 mg·kg^−1^ from El Oro province, while the minimum was 0.55 mg·kg^−1^ from the province of Los Ríos. In soils from Santa Elena (which was part of Guayas province at the time the study was conducted), the Pb concentrations were near 2 mg·kg^−1^. As Felix et al. [44] mentioned, major concentrations of toxic metals in soils allows for the metals to be taken up by plants, but this also depends on the production areas’ proximity to roads and possible sources of hydrocarbon contamination.

In the Los Ríos, El Oro, and Guayas provinces, the RSD between samples were also calculated, resulting in RSDs of 26.15% (three samples), 0.28% (two samples), and 19.31% (three samples), respectively. We assume that this variability in the sample results is related to external factors, namely, the contaminants to which each production location is exposed.

Other international studies have estimated Cd and Pb concentrations and used them for risk assessment calculations, as shown in Table 2. The results obtained in the present study are consistent with those from other studies. For Cd, the mean concentration of 24.0 μg·kg^−1^ was within the value range reported for bananas in Serbia (<0.3 μg·kg^−1^) [9] and Jamaica (57.0 μg·kg^−1^) [3]. In the case of Ni, the concentration obtained (29.0 μg·kg^−1^) was lower than samples from Bangladesh (37.0 μg·kg^−1^) [1]. For Pb content, the mean result was 192.0 μg·kg^−1^, higher than many other countries, but four times lower than the concentration in Nigerian samples (460.0 µg·kg^−1^) [45].

### 3.3. Quality Control

Blanks, triplicates of non-fortified samples, and each sample’s fortifications were used as quality controls.

The RSD values obtained were lower than 7.77% for Cd, lower than 6.87% for Ni, and lower than 3.74% for Pb, in accordance with the recommended AOAC (2002) value of 8% for repeatability RSD [35]. 

Regarding accuracy, the results obtained were between 82.90% and 119.78%, between 80.63% and 116.60%, and between 81.29% and 115.31% for Cd, Ni, and Pb, respectively. All the accuracy values were within the AOAC (2002) accuracy recommendations of between 75.00% and 120.00%.

### 3.4. Health Risk Assessment Results

**Figure 1:**
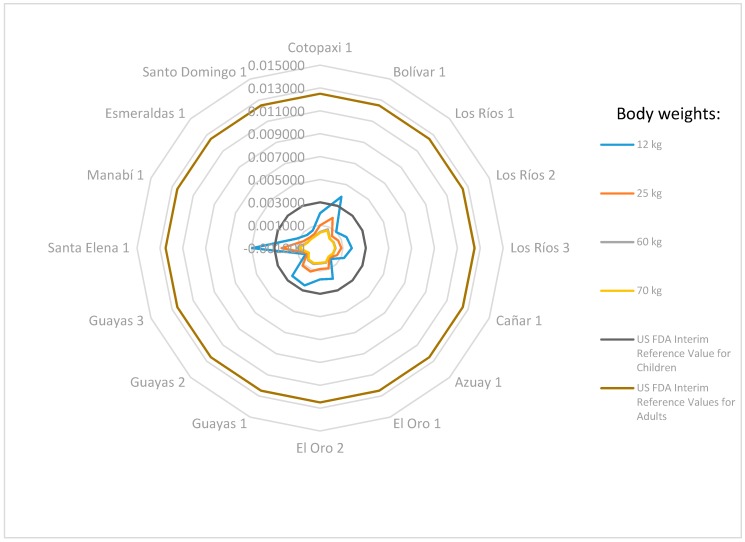
Lead exposure values calculated with sample metal concentrations (µg·kg^−1^·day^−1^) compared with the oral reference dose (RfD) of each metal that could be safely consumed daily (µg·kg^−1^·day^−1^) for different body weights (kg).

EDI values were calculated for different body weights (12, 25, 60, and 70 kg) for all the samples analyzed, and the results of Cd, Ni, and Pb were between 0.015 µg·kg^−1^·day^−1^ and 0.434 µg·kg^−1^·day^−1^, between 0.025 µg·kg^−1^·day^−1^ and 0.968 µg·kg^−1^·day^−1^, and between 0.058 µg·kg^−1^·day^−1^ and 4.932 µg·kg^−1^·day^−1^, respectively. In Cd and Ni cases, the results were below the EPA RfD references of 1.000 µg·kg^−1^·day^−1^ and 20.0 µg·kg^−1^·day^−1^, respectively [24]. In the case of Pb, two results were higher than 3.0 µg·kg^−1^·day^−1^ (US FDA interim reference values for toddlers and children), and these values were 3.858 and 4.932 µg·kg^−1^·day^−1^ in the Bolivar and Santa Elena samples, respectively, for toddlers (12 kg BW). In the case of children (25 kg BW) and adults (60 and 70 kg BW), all results were lower than 3.0 and 12.5 µg·kg^−1^·day^−1^ reference values (US FDA), respectively [25]. The Cd and Ni exposures are quite low than the RfD values, in both cases. The lead results are shown in Figure 1.

For the calculated THQ values, the results were between 0.015 and 0.434 for Cd, between 0.002 and 0.088 for Ni, and between 0.019 and 1.644 for Pb. All the THQ values for Cd and Ni were under the established criteria, but in the case of Pb, for toddlers (12 kg BW), the THQ of the Bolivar (1.286) and Santa Elena (1.644) province samples were higher than 1. This fact is also evident with the EDI results, higher than the US FDA interim reference values for children of 3.0 µg·kg^−1^·day^−1^.

The TTHQ results were obtained from the sum of each metal’s THQ corresponding to each body weight and sample location. The highest value obtained for all the body weights were for the Santa Elena province. The results were 1.85, 0.89, 0.37, and 0.32 for 12, 25, 60, and 70 kg, respectively.

The CR was determined for all the samples. For the Pb results, 93.75%, and 37.50% of the sample results for the body weights of 12 and 25 kg, respectively, were higher than 10^−6^ but lower than 10^−4^. These results are within the acceptable range as no samples were higher than the intolerable limit of 10^−4^ [41,42]. 

## 4. Conclusions

This study determined the concentrations of three toxic elements in bananas produced and commercialized in Ecuador, which are among Ecuador’s most exported products. The samples analyzed do not present a non-carcinogenic risk for human health in the cases of Cd and Ni. For Pb every result was within the acceptable range for the carcinogenic risk, but two samples presented THQ values higher than the US FDA Interim Reference Values in the calculations for toddlers (12 kg BW). These results show that exposure to Pb deserves particular attention, principally in the case of samples from the Bolivar and Santa Elena provinces, where banana consumption by toddlers could pose a potential health risk .

Comparison with available international studies focusing on risk assessment for toxic metal intake revealed differences among the concentrations of these contaminants in the samples analyzed in the present study. This result could be mainly attributed to variations in the natural compositions of soils as well as to differences in consumers’ dietary habits. 

Future investigations are necessary to estimate the THQs more accurately for the three metals, and the CR for Pb, to understand the probability of ingesting levels of these metals in which its concentrations are above the safe thresholds, letting to establish a more comprehensive view of the safety of Ecuadorian food products.

## Figures and Tables

**Table 1:** Results summary of concentrations in original samples (Cc, μg·kg^−1^), rates of standard deviation (RSD, %), and “in-house” accuracy (%) of cadmium (Cd), nickel (Ni), and lead (Pb) determinations in Ecuadorian banana samples and the threshold values (μg·kg^−1^).

Provinces	Samples	Cd			Ni			Pb		
		Cc	RSD	Accuracy	Cc	RSD	Accuracy	Cc	RSD	Accuracy
Cotopaxi	Cotopaxi 1	9.3	5.54%	95.22%	105.6	1.54%	107.31%	**224.4**	0.33%	93.50%
Bolívar	Bolívar 1	32.5	5.38%	110.34%	27.7	5.93%	101.29%	**420.9**	1.88%	114.62%
Los Ríos	Los Ríos 1	20.1	7.73%	93.50%	23.2	3.61%	105.90%	**107.7**	3.27%	93.72%
	Los Ríos 2	19.4	5.26%	95.73%	29.1	6.16%	83.96%	**163.0**	2.26%	115.31%
	Los Ríos 3	30.2	5.93%	96.52%	21.5	6.87%	112.57%	**193.5**	2.63%	106.29%
Cañar	Cañar 1	33.5	6.68%	119.78%	21.8	6.32%	80.63%	**138.1**	0.78%	108.48%
Azuay	Azuay 1	26.0	5.55%	88.49%	29.8	0.83%	107.39%	36.9	0.81%	109.31%
El Oro	El Oro 1	29.8	5.39%	92.73%	24.9	6.40%	109.60%	**208.2**	3.47%	86.55%
	El Oro 2	29.6	1.17%	86.47%	24.5	5.34%	87.06%	**189.3**	0.56%	82.68%
Guayas	Guayas 1	17.4	7.43%	105.97%	30.5	6.87%	108.01%	**276.5**	3.74%	111.64%
	Guayas 2	24.3	7.77%	87.96%	22.3	5.58%	116.60%	**266.5**	1.18%	83.36%
	Guayas 3	17.9	5.95%	101.74%	21.6	5.74%	116.36%	40.1	3.16%	88.98%
Santa Elena	Santa Elena 1	21.3	7.19%	82.90%	17.7	4.31%	85.99%	**538.0**	1.81%	84.27%
Manabí	Manabí 1	47.3	4.07%	97.19%	30.8	4.84%	84.89%	**128.8**	2.93%	81.29%
Esmeraldas	Esmeraldas 1	13.8	6.48%	86.64%	16.1	5.77%	96.12%	67.0	3.74%	81.96%
Santo Domingo	Santo Domingo 1	14.9	4.62%	107.59%	19.4	4.38%	95.89%	72.9	0.51%	109.04%
Threshold values		50.0 ^a^	-	-	300.0 ^b^	-	-	100.0 ^a,c^	-	-

^a^ European Commission. Commission Regulation No 1881/2006 of 19 December 2006 [21]. **^b^** EPA-IRIS. Nickel, soluble salts; CASRN Various. Integrated Risk Information System (IRIS) Chemical Assessment Summary [18]. ^c^ FAO/WHO General Standard for Contaminants and Toxins in Food and Feed CXS 193-1995 (Revision 2018) [22].

**Table 2:** Comparison among similar studies done, a summary of cadmium (Cd), nickel (Ni), and lead (Pb) mean concentrations (μg·kg^−1^), the estimated daily intakes (EDI, µg·kg^−1^·day^−1^), target hazard quotients (THQ, unitless), and carcinogenic risks (CR, unitless).

Country	Metal	Mean concentrations	EDI	THQ	CR	References
Bangladesh	Cd	ND	NA	0	NA	[1]
	Ni	37.0	2.80 × 10^−5^	1.00 × 10^−3^	NA	
	Pb	3.0	2.20 × 10^−6^	6.00 × 10^−4^	1.90 × 10^−8^	
Serbia	Cd	<0.3	0.002	NA	NA	[9]
	Pb	60.0	1.254	NA	NA	
Jamaica	Cd	57.0	0.028	0.028	NA	[3]
	Pb	10.0	0.005	0.002	NA	
Nigeria	Cd	ND	NA	NA	NA	[45]
	Pb	460.0	0.0028	NA	NA	
Ecuador *	Cd	24.0	4.44 × 10^−5^	0.044	NA	The present study
	Ni	29.0	5.35 × 10^−5^	0.005	NA	
	Pb	192.0	3.52 × 10^−4^	0.099	3.77 × 10^−7^	

ND: Not detectable, NA: Not available. * Mean values for 60 kg of body weight (generally used by the WHO for calculation of Acceptable Daily Intakes (ADIs) and adopted in the work of some EFSA Panels [36].
